# The CHA_2_DS_2_-VASc Score Predicts Major Bleeding in Non-Valvular Atrial Fibrillation Patients Who Take Oral Anticoagulants

**DOI:** 10.3390/jcm7100338

**Published:** 2018-10-09

**Authors:** Kuang-Tso Lee, Shang-Hung Chang, Yung-Hsin Yeh, Hui-Tzu Tu, Yi-Hsin Chan, Chi-Tai Kuo, Lai-Chu See

**Affiliations:** 1 Cardiovascular Department of Chang-Gung Memorial Hospital, Linkou, Taoyuan City 33305, Taiwan; Hornkimq@gmail.com (K.-T.L.); yeongshinn@cgmh.org.tw (Y.-H.Y.); s851047@hotmail.com (Y.-H.C.); chitai@cgmh.org.tw (C.-T.K.); 2 Center for Big Data Analytics and Statistics, Chang Gung Memorial Hospital, Linkou, Taoyuan City 33305, Taiwan; tzujob@gmail.com; 3 Medical School, Chang Gung University, Taoyuan City 33302, Taiwan; 4 Graduate Institute of Nursing, Chang Gung University of Science and Technology, Taoyuan City 33303, Taiwan; 5 Department of Public Health, College of Medicine, Chang Gung University, Taoyuan City 33305, Taiwan; 6 Biostatistics Core Laboratory, Molecular Medicine Research Center, Chang Gung University, Taoyuan City 33305, Taiwan; 7 Division of Rheumatology, Allergy and Immunology, Department of Internal Medicine, Chang Gung Memorial Hospital, Linkou, Taoyuan City 33305, Taiwan

**Keywords:** atrial fibrillation, CHA_2_DS_2_-VASc score, oral anticoagulant, warfarin

## Abstract

Background: Patients with atrial fibrillation (AF) are at a substantial risk of ischemic stroke. The CHA_2_DS_2_-VASc score predicts the risk of thromboembolism, but its role in predicting major bleeding in patients taking oral anticoagulants is unclear. Methods: We used the National Health Insurance Research Database (NHIRD) of Taiwan to identify patients with AF from 2010 to 2016. They were divided into four groups according to the oral anticoagulants. The outcomes were ischemic stroke/systemic thromboembolism, and major bleeding. Results: A total of 279,776 patients were identified. Ischemic stroke or systemic embolism events were observed in 1.73%, 3.62%, 4.36%, and 5.02% of the patients in the apixaban, rivaroxaban, dabigatran, and warfarin groups, respectively. Major bleeding was recorded in 1.18%, 2.66%, 3.23%, and 4.70% of the patients in the apixaban, rivaroxaban, dabigatran, and warfarin groups, respectively. The highest rates for both ischemic stroke and bleeding events occurred in the patients with a CHA_2_DS_2_-VASc score of five or more. Conclusion: Non-valvular AF patients with high CHA_2_DS_2_-VASc scores are susceptible to both systemic thromboembolism and major bleeding. The trend was consistently observed in patients who took non-vitamin K oral anticoagulants (NOACs) or warfarin. NOACs might be potentially more effective in reducing overall events.

## 1. Introduction

Patients with atrial fibrillation (AF) are at substantial risk of ischemic stroke and systemic embolism. Oral anticoagulation therapy has been shown to be effective in preventing thromboembolic complications [1,2,3,4,5,6]. However, such therapy has also been shown to increase the risk of major bleeding [3,7,8,9]. Therefore, the benefits of treatment should be weighed against the risks.

To estimate the incidence of systemic embolism and major bleeding in patients with AF, an evidence-based scoring system is needed. The CHADS_2_ score is one of the most studied models [10], and it has been used to estimate the risk of ischemic stroke and systemic embolism. The subsequently released CHA_2_DS_2_-VASc score contains more parameters [11], and its use is recommended in current guidelines [12,13].

In general, non-valvular AF patients with higher CHA_2_DS_2_-VASc scores are considered to be more susceptible to thromboembolic events. Warfarin and non-vitamin K oral anticoagulants (NOACs) such as dabigatran, rivaroxaban, and apixaban have been shown to be effective in reducing the risk of ischemic stroke and systemic embolism [2,3,4]. In addition, current guidelines recommend that the risk of bleeding is assessed before initiating oral anticoagulant (OAC) therapy [12,13]. Several scoring systems such as the HEMORR_2_HAGES, HAS-BLED, ATRIA, ORBIT, and ABC systems have been used to predict the risk of bleeding for patients under OAC treatment [14,15,16,17,18].

Although it has been reported that higher CHA_2_DS_2_-VASc scores are also associated with an increased incidence of major bleeding in patients receiving warfarin or rivaroxaban [19,20], it is not known whether this phenomenon would be observed in patients taking other NOACs such as dabigatran and apixaban. In this study, we investigated this issue using a nationwide medical insurance claiming database in Taiwan.

## 2. Materials and Methods

### 2.1. Patients

**Figure 1:**
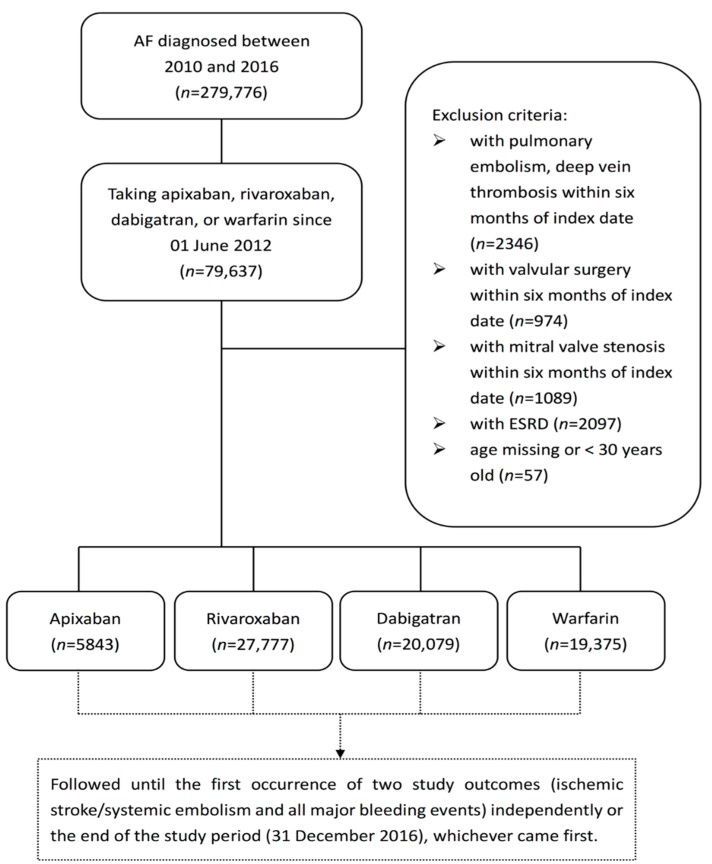
Study flowchart. AF = atrial fibrillation, ESRD = end-stage renal disease.

We used the National Health Insurance Research Database (NHIRD) of Taiwan to analyze patients between 2010 and 2016 with a diagnosis of AF. The International Statistical Classification of Disease and Related Health Problems, Ninth Revision, Clinical Modification (ICD-9-CM) code 427.31 and International Statistical Classification of Disease and Related Health Problems, Tenth Revision, Clinical Modification (ICD-10-CM) code 148 were used to select AF patients. A total of 279,776 cases were identified. Those not prescribed with warfarin or any of the three NOACs (dabigatran, rivaroxaban, and apixaban), and those who had ever used more than one of these NOACs were excluded. The other major exclusion criteria were the occurrence of pulmonary embolism, valvular surgery, or hemodialysis within 6 months before the index date, defined as the date of the first warfarin or NOAC prescription. The flow chart of subject enrollment is shown in Figure 1. We divided the target patients into four groups: apixaban (5843 patients, 8.00%), rivaroxaban (27,777 patients, 38.01%), dabigatran (20,079 patients, 27.48%), and warfarin (19,375 patients, 26.51%). All comorbidities in Table 1 were defined as events leading to at least two outpatient visits and/or hospitalization at least once. The CHA_2_DS_2_-VASc score contains seven categories: congestive heart failure, hypertension, age, diabetes mellitus, stroke, vascular disease, and sex. ICD-9-CM and ICD-10-CM codes were used to identify these diseases. Because some drugs, such as anti-platelet agents and Non-steroidal anti-inflammatory drugs (NSAIDs), reduce the occurrence of bleeding, they were considered as well.

### 2.2. Outcomes

The study outcomes were major bleeding, ischemic stroke, and systemic embolism. Major bleeding was defined as intracranial bleeding, gastrointestinal bleeding, and any bleeding events in patients who needed hospitalization. Only outcomes which occurred after the index date were counted.

### 2.3. Statistical Methods

Incidence rates were computed using the total number of study outcomes during the follow-up period divided by person-years at risk. Cumulative incidence rates were also computed by the total number of study outcomes during the follow-up period divided by persons involved at the index date. ANOVA or the chi-squared test was used to compare data among the four groups, where appropriate. Statistical significance was defined as a *p* value < 0.05. All statistical analyses were performed using SAS 9.2 (SAS Institute Inc., Cary, NC, USA).

## 3. Results

**Figure 2:**
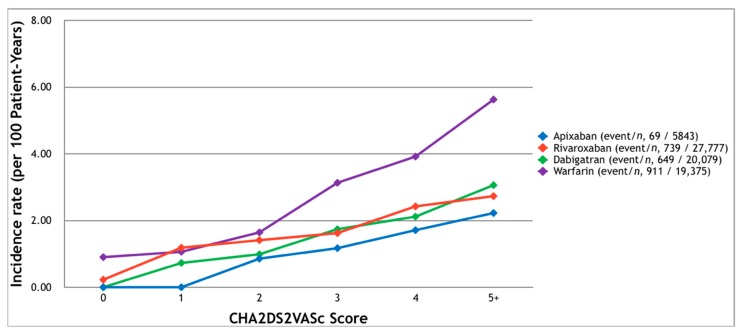
The incidence of major bleeding which required hospitalization among patients with non-valvular atrial fibrillation and receiving oral anticoagulants. Major bleeding occurred more frequently in patients with higher CHA_2_DS_2_-VASc scores.

The baseline characteristics of the studied patients are listed in Table 1. Major bleeding events were recorded in 69, 739, 649, and 911 patients in the apixaban, rivaroxaban, dabigatran, and warfarin groups, respectively (Figure 2). The cumulative incidence rates of major bleeding in 2.5 years were 1.18%, 2.66%, 3.23%, and 4.70%, respectively. Except for the patients with a CHA_2_DS_2_-VASc score of one in the warfarin group, the events occurred more frequently in the patients with higher scores, especially scores of five or higher (2.23%, 3.06%, 2.73%, and 5.63%, respectively).

**Figure 3:**
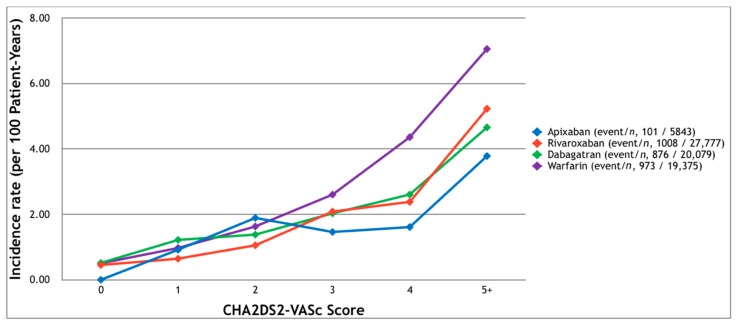
The incidence of ischemic stroke or systemic emboli requiring hospitalization among patients with non-valvular atrial fibrillation receiving oral anticoagulants. Ischemic stroke and systemic emboli occurred more frequently in the patients with higher CHA_2_DS_2_-VASc scores.

**Figure 4:**
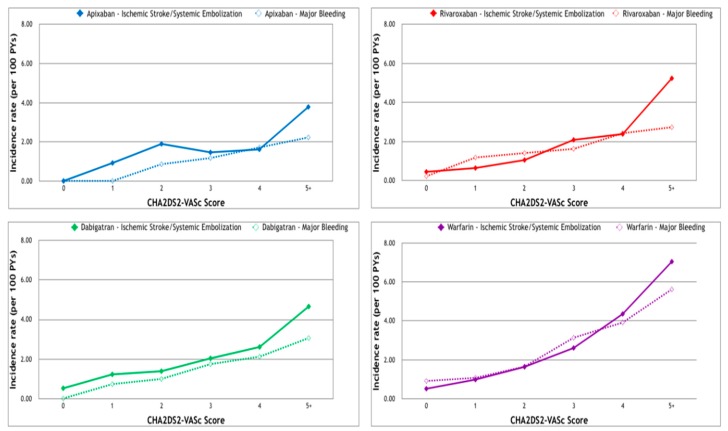
Incidence of major bleeding and ischemic stroke/systemic emboli requiring hospitalization among patients with non-valvular atrial fibrillation receiving oral anticoagulants. Upper left: the apixaban group. Upper right: the rivaroxaban group. Lower left: the dabigatran group. Lower right: the warfarin group.

Ischemic stroke or systemic embolism was observed in 101, 1008, 876, and 973 patients in the apixaban, rivaroxaban, dabigatran, and warfarin groups, respectively (Figure 3), corresponding to cumulative incidence rates of 1.73%, 3.62%, 4.36%, and 5.02%. As the CHA_2_DS_2_-VASc score increased, more events were noted in all but the apixaban group (with a score of three). The highest event rate was seen in the patients with a CHA_2_DS_2_-VASc score of five or more (3.78%, 5.23%, 4.66%, and 7.06% in the apixaban, rivaroxaban, dabigatran, and warfarin groups, respectively). In all four groups, where there was a CHA_2_DS_2_-VASc score of five or more, the risk of stroke or systemic embolism was higher than the risk of major bleeding (Figure 4).

## 4. Discussion

Warfarin has been considered a standard treatment in the prevention of atrial fibrillation-related thromboembolism. Previous studies proved that the patients receiving NOACs enjoyed similar preventive effects and a lower risk of major bleeding [12,13,21]. Efficacy and safety have also been highlighted in electrical cardioversion and transcatheter radiofrequency cardiac ablation [22,23,24]. The evidence supported that NOACs would be some of the first drug choices in the treatment of non-valvular atrial fibrillation. To further stratify the benefit and the risk of the patients under the treatment of different NOACs, we conducted this large-population study. It revealed a correlation between CHA_2_DS_2_-VASc score and major bleeding in non-valvular atrial fibrillation (NVAF) patients. We found that the patients with higher CHA_2_DS_2_-VASc scores were more susceptible to both major bleeding and systemic thromboembolism.

Current guidelines suggest the use of CHA_2_DS_2_-VASc scores to estimate the risk of ischemic stroke for non-valvular AF patient and initiate oral anticoagulant therapy accordingly (a score of two for male or more for female patients). To reduce the incidence of bleeding, the guidelines also recommend using scoring systems such as the HAS-BLED, ORBIT, or ABC scores, to identify patients at high risk of bleeding. However, the benefits of thromboembolic prevention should be weighed against the risk of major bleeding [12,13].

An association between CHA_2_DS_2_-VASc scores and the incidence of major bleeding has been reported in several previous studies [19,20], in which a higher risk of bleeding was noted in patients with higher CHA_2_DS_2_-VASc scores who were also receiving warfarin or rivaroxaban. We also found a similar association in the patients taking dabigatran or apixaban. This finding should serve to remind clinicians that AF patients at high risk of thromboembolic events are also at a considerably higher risk of bleeding. This may be because these two different types of complications share similar risk factors. 

There are several possible explanations why the CHA_2_DS_2_-VASc score may predict major bleeding. For example, the CHA_2_DS_2_-VASc and HAS-BLED scores share similar risk factors, including hypertension, old age, and history of prior stroke [11,15]. The CHA_2_DS_2_-VASc score includes diabetes mellitus, which has a high incidence in patients with chronic kidney disease, which in turn is a component of the HAS-BLED score [25]. The associations among coronary artery disease, peripheral artery disease, heart failure, and major bleeding may be more complicated. Patients with coronary or peripheral artery disease receive long-term anti-platelet therapy, which can increase the risk of bleeding [26,27,28]. The prevalence of coronary or peripheral artery diseases is higher in patients with chronic kidney disease than in the general population [29]. Heart failure patients are also susceptible to ischemic heart disease, peripheral artery disease, and chronic kidney disease [30,31]. Therefore, it is reasonable that the CHA_2_DS_2_-VASc score could predict the risk of both thromboembolism and major bleeding.

There is a relatively high proportion of using anti-platelet agents in our study groups. It is reasonable to see the high proportion of the combination therapy to prevent bleeding among patients with acute coronary syndrome and coronary artery stenting. On the other hand, the prevalence of ischemic heart disease is not compatible with the proportion of anti-platelet, we guess that such prescription was not prolonged. Because the rate of major bleeding in this study is similar to the previous report in the United States, the predictability of the CHA_2_DS_2_-VASc score on bleeding is not affected [32]. Nevertheless, the effect of combination of anti-platelets and oral anticoagulants on bleeding deserves further researches.

Our results showed that the patients in the Warfarin group had the highest prevalence rates of systemic thromboembolism and major bleeding, especially with a CHA_2_DS_2_-VASc score of four or more. Similar results have also been reported in other studies, especially in Asian populations. Possible explanations include labile international normalized ratio and specific genetic polymorphisms [33,34,35]. These results highlight again that warfarin is not the best choice to treat Asian AF patients.

## 5. Limitations

There are a several limitations to this study. First, we did not compare CHA_2_DS_2_-VASc scores with other risk scores for their ability to predict major bleeding. Several scoring systems are available to estimate the risk of bleeding, such as the HAS-BLED, ORBIT, or ABC scores. Though the components of the CHA_2_DS_2_-VASc score have been well-documented in the NHIRD, some of the factors in other scoring systems could not be accurately extracted from the database. As in some other studies using national health insurance data, Taiwan’s NHIRD does not include echocardiographic parameters such as ejection fraction of left ventricle, left atrial size, or right ventricular systolic function. Second, the weighting of individual risk categories in the CHA_2_DS_2_-VASc score was not well defined. Hence, we did not know which risk factors were more associated with thromboembolism or major bleeding. Third, the sample size apixaban group was relatively small because apixaban has been approved for a shorter period of time. Edoxaban, another NOAC, was not included in this study for the same reason. Researchers may wish to study these two NOACs further in the future.

## 6. Conclusions

Non-valvular AF patients with high CHA_2_DS_2_-VASc scores are susceptible to both major bleeding and systemic thromboembolism. Oral anticoagulants should be prescribed carefully for patients with high CHA_2_DS_2_-VASc scores to avoid adverse bleeding events. The outcomes of the patients with lower CHA_2_DS_2_-VASc scores were similar in the warfarin and the NOAC groups, but the NOACs were potentially more effective at reducing overall events in patients with high CHA_2_DS_2_-VASc scores. The threshold of the CHA_2_DS_2_-VASc score in terms of net benefit might be higher for NVAF patients in Taiwan.

## Figures and Tables

**Table 1:** Demographic, comorbidities, and medication used among four study groups.

	Apixaban (*n* = 5843)	Rivaroxaban (*n* = 27,777)	Dabagatran (*n* = 20,079)	Warfarin (*n* = 19,375)	*p*-Value
Demographics					
Age (year)	76 ± 10	75 ± 10	75 ± 10	71 ± 13	<0.0001
Male gender	3214 (55.01%)	15,374 (55.35%)	12,061 (60.07%)	11,221 (57.91%)	<0.0001
CHA_2_DS_2_-VASc score	3.89 ± 1.56	3.83 ± 1.57	3.74 ± 1.52	3.26 ± 1.81	<0.0001
Comorbidities					
Hypertension	5055 (86.51%)	23,766 (85.56%)	16,863 (83.98%)	15,099 (77.93%)	<0.0001
Diabetes mellitus	2389 (40.89%)	10,752 (38.71%)	7647 (38.08%)	6948 (35.86%)	<0.0001
PAOD	4 (0.07%)	19 (0.07%)	11 (0.05%)	16 (0.08%)	0.7736
Ischemic heart disease	733 (12.54%)	3399 (12.24%)	1961 (9.77%)	2098 (10.83%)	<0.0001
PCI	415 (7.1%)	1750 (6.3%)	916 (4.56%)	1051 (5.42%)	<0.0001
CABG	31 (0.53%)	104 (0.37%)	40 (0.2%)	143 (0.74%)	<0.0001
Heart failure	735 (12.58%)	3582 (12.9%)	2172 (10.82%)	2699 (13.93%)	<0.0001
Chronic kidney disease	1671 (28.6%)	6786 (24.43%)	3922 (19.53%)	4702 (24.27%)	<0.0001
Chronic liver disease	929 (15.9%)	4421 (15.92%)	2831 (14.1%)	3048 (15.73%)	<0.0001
TIA	167 (2.86%)	667 (2.4%)	573 (2.85%)	344 (1.78%)	<0.0001
Stroke	1173 (20.08%)	5675 (20.43%)	4778 (23.8%)	2936 (15.15%)	<0.0001
History of bleeding	113 (1.93%)	644 (2.32%)	415 (2.07%)	451 (2.33%)	0.0841
Medication used					
NSAID	1556 (26.63%)	6657 (23.97%)	4401 (21.92%)	4792 (24.73%)	<0.0001
Anti-platelets	3231 (55.3%)	15,450 (55.62%)	10,906 (54.32%)	11,907 (61.46%)	<0.0001
H_2_ blocker	1810 (30.98%)	8175 (29.43%)	5772 (28.75%)	6200 (32%)	<0.0001
PPI	655 (11.21%)	2906 (10.46%)	1654 (8.24%)	2421 (12.5%)	<0.0001
Amiodarone	1649 (28.22%)	7370 (26.53%)	4498 (22.4%)	7472 (38.57%)	<0.0001
Beta-blocker	3451 (59.06%)	15,782 (56.82%)	10,839 (53.98%)	11,824 (61.03%)	<0.0001
Statin	229 (3.92%)	3949 (14.22%)	4101 (20.42%)	3322 (17.15%)	<0.0001

PAOD = peripheral arterial occlusive disease, PCI = percutaneous coronary intervention, CABG = coronary artery bypass graft, TIA = transient ischemic attack, NSAID = non-steroidal anti-inflammatory drug, PPI = proton pump inhibitor.
